# VJ Segment Usage of TCR-Beta Repertoire in Monozygotic Cystic Fibrosis Twins

**DOI:** 10.3389/fimmu.2021.599133

**Published:** 2021-02-23

**Authors:** Sebastian Fischer, Frauke Stanke, Burkhard Tümmler

**Affiliations:** ^1^Clinic for Pediatric Pneumology, Allergology and Neonatology, Hannover Medical School, Hannover, Germany; ^2^Biomedical Research in Endstage and Obstructive Lung Disease Hannover (BREATH), German Center for Lung Research, Hannover Medical School, Hannover, Germany

**Keywords:** T cell receptor repertoire, immunotyping, VJ usage, cyctic fibrosis, twins, CDR3, TCRB

## Abstract

Sixteen monozygotic cystic fibrosis (CF) twin pairs of whom 14 pairs were homozygous for the most common p.Phe508del *CFTR* mutation were selected from the European Cystic Fibrosis Twin and Sibling Study Cohort. The monozygotic twins were examined in their T cell receptor (TCR) repertoire in peripheral blood by amplicon sequencing of the CDR3 variable region of the ß-chain. The recruitment of TCR J and V genes for recombination and selection in the thymus showed a strong genetic influence in the CF twin cohort as indicated by the shortest Jensen-Shannon distance to the twin individual. Exceptions were the clinically most discordant and/or most severely affected twin pairs where clonal expansion probably caused by recurrent pulmonary infections overshadowed the impact of the identical genomic blueprint. In general the Simpson clonality was low indicating that the population of TCRß clonotypes of the CF twins was dominated by the naïve T-cell repertoire. Intrapair sharing of clonotypes was significantly more frequent among monozygotic CF twins than among pairs of unrelated CF patients. Complete nucleotide sequence identity was observed in about 0.11% of CDR3 sequences which partially should represent persisting fetal clones derived from the same progenitor T cells. Complete amino acid sequence identity was noted in 0.59% of clonotypes. Of the nearly 40,000 frequent amino acid clonotypes shared by at least two twin siblings 99.8% were already known within the immuneACCESS database and only 73 had yet not been detected indicating that the CDR3ß repertoire of CF children and adolescents does not carry a disease-specific signature but rather shares public clones with that of the non-CF community. Clonotypes shared within twin pairs and between unrelated CF siblings were highly abundant among healthy non-CF people, less represented in individuals with infectious disease and uncommon in patients with cancer. This subset of shared CF clonotypes defines CDR3 amino acid sequences that are more common in health than in disease.

## Introduction

Cystic fibrosis (CF) is a systemic disorder of exocrine glands that is caused by mutations in the *Cystic Fibrosis Transmembrane Conductance Regulator (CFTR)* gene (1, 2). The basic defect manifests in an impaired luminal transport of chloride and bicarbonate leading in the lungs to compromised innate immunity, airway obstruction by mucus plugging and inhomogeneous ventilation (3). Chronic airway infections are the major cause for morbidity and mortality of this life-limiting autosomal recessive trait. Triggered by recurrent viral infections, the upper and lower airways are colonized with bacterial opportunistic pathogens, namely *Staphylococcus aureus* and – later in life – *Pseudomonas aeruginosa* (4). Bacterial persistence in biofilms and immigration of immune effector cells sustain a vicious cycle of chronic infection, inflammation, lung damage and remodeling which leads to ongoing deterioration of pulmonary function and finally to respiratory failure (5).

Massive neutrophil influx and subsequent release of neutrophil extracellular traps are key events that result in the accumulation of extracellular DNA and enzymes such as elastase and myeloperoxidase causing persistent CF lung injury (6). Neutrophilic infiltration of the airways occurs secondary to the presence of cytokines and chemokines. Notably, early signs of lung inflammation can precede colonization and infection suggesting that inflammation caused by mucus plugging of airways is the primary event in CF lung disease.

T lymphocytes with appropriate antigenic specificity to luminal pathogens are present in high numbers in the CF airway submucosa, alveolar septa, and draining lymph nodes, but there is a striking paucity of T lymphocytes in the lumen itself (7, 8). Neutrophils and T lymphocytes are compartmentalized in CF airways, with neutrophils accumulating in the lumen, whereas T cells stay in the submucosa and lymph nodes and are excluded from the lumen. Enhanced granule release from the neutrophils, the concomitant release of arginase and pro-apoptotic factors in the CF airway fluid are probably responsible for this exclusion/early blockade of T cell expansion in the CF airway lumen (9). This dysregulation of the T cell response may play an important role in the virus-related exacerbation of bacterial infections in the CF airways (6).

The T-cell receptor (TCR) on the surface of T lymphocytes recognizes fragments of antigen as peptides bound to major histocompatibility complex (MHC) molecules (10). T lymphocytes mature from thymus cells called thymocytes (11, 12). Each thymocyte has a distinct T-cell receptor. Thymocytes will die by apoptosis if their T-cell receptor reacts too weakly or not at all with a peptide/MHC ligand of the epithelial cells in the cortex or too strongly to self antigens in the medulla of the thymus (11–15). Less than one percent of thymocytes survive the positive and negative selection in the thymus and emigrate into the periphery as naïve T cells where during an infection the high-affinity recognition of an antigen on a MHC molecule will initiate an immune response and the clonal T cell proliferation.

TCRs are heterodimers, composed for most T cells of an α- and a β-chain with each T cell clone expressing a unique combination. Peptide specificity of αβ T cells is primarily determined by the amino acid sequence encoded in the third complementarity-determining region (CDR3) loops of the α- and β-chain variable domains. In the ß-chain locus the CDR3 regions are formed by somatic recombination of paralogous variable (V), diversity (D) and joining (J) gene segments, with additional removal and random addition of nucleotides at the V-J, V-D and D-J junctions thus permitting an enormous combinatorial diversity in receptor composition (16). We were curious to know how the inherited CF condition affects the TCR profiles that are selected from a diverse, naive repertoire.

Considering the magnitude of theoretically possible diversity of 10^18^ TCR α-β pairs (17), the sharing of TCR sequences among individuals should be very rare. The frequency of α-β pairs has indeed been shown to be a stochastic product of individual germline encoded V and J gene segment usage (18). However, a subset of so-called “public” TCR sequences is frequently shared between individuals (19, 20). Studies on the TCR repertoire in monozygotic twins moreover identified a genetic influence on sharing of identical TCR sequences (18, 21–24) occurring at a normalized rate of 10^−7^ (21).

These studies on healthy twin pairs suggest that genetic determinants impose constraints on the human TCR repertoire. Cystic fibrosis is the most common monogenic disorder in Caucasian populations that predisposes to chronic life-limiting infection. This inherited disease offers the opportunity to examine the constraints on the TCR repertoire in a compromised host susceptible to infection who does not harbor an inborn error in the innate or adaptive immune system. Thanks to the support of the European Cystic Fibrosis Society we could recruit and examine monozygotic twins with cystic fibrosis who are homozygous for the most common disease-causing *CFTR* mutation p.Phe508del (25).

Here we report on the sequence diversity of the antigen-binding *TCRß* CDR3 region in peripheral blood of 16 twin pairs, the data of which have already been communicated in another context for three pairs (26). Monozygotic twins have identical genes encoding the MHC I alleles and the TCR recombination machinery. Hence, based on data published for healthy twins (18, 21, 23, 24) we expected a more similar distribution of V and J segments in a CF twin pair than between unrelated CF individuals. This assumption turned out to be true for the majority of twin pairs but a minority showed a more individual expansion of segments in their TCR repertoire.

## Materials and Methods

### European Cystic Fibrosis Twin and Sibling Study Cohort (25)

530 CF patient pairs were enrolled from 158 CF clinics in 14 European countries who provided information about date of birth, gender, zygosity status of twins, and the most recent height, weight and spirometry (FEV1, forced expiratory volume in 1 s). The anthropometric parameter wfh%, percentage weight for height predicted, was calculated from the comparison of the measured height and weight to the predicted weight of a healthy individual of the same height, age and gender extracted from the centile distribution of the Zürich study on growth and development (27). To correct for the CF-specific decline of FEV1%pred with age, CF specific disease centiles for FEV1, called FEVPerc, were determined by mapping the patient's FEV1%pred referring to a non-CF population (28) onto the age- and gender-dependent centile distributions of FEV1%pred based on lung function data of 25,667 European CF patients compiled in the ERCF Annual Report 1998 (29).

The complete cohort of 1,060 patients was sorted by rank numbers x_i_ for wfh% and y_i_ for the FEV1 disease centile FEVPerc, whereby the rank 1 indicated the most severely affected state.

By giving the rank numbers for anthropometry and lung function the same weight, the CF disease severity of an individual *i* in the cohort was defined by

$$\begin{array}{l} {\left(x_{\text{i}}^{2}+y_{\text{i}}^{2}\right)}^{1/2} \end{array}$$

and the intrapair discordance Δ between the two individuals *i* and *j* of a twin and sibpair by

$$\begin{array}{l} \Delta ={\left[\left(x_{\text{i}}^{2}-x_{\text{j}}^{2}\right)+\left(y_{\text{i}}^{2}-y_{\text{j}}^{2}\right)\right]}^{1/2}. \end{array}$$

Rank numbers for Δ were assigned to the complete twin and sibpair cohort, whereby the rank 1 indicated the most concordant pair.

The reported zygosity status of 41 twin pairs was tested by multiplex PCR of nine short tandem repeat loci (D3S1358, D5S818, D7S820, D8S1179, D13S317, D18S51, D21S11, FGA, and vWA) (30).

### Monozygotic Twins

Sixteen monozygotic twin pairs were recruited for the investigation of the TCR β-chain diversity of whom DNA or blood for DNA extraction, clinical data and in-house verified information about *CFTR* genotype and zygosity status were available. Fourteen exocrine pancreatic insufficient pairs of non-consanguineous marriage are homozygous for the most common CF causing mutation p.Phe508del (31). As non-congruent outliers of *CFTR* genotype two further pairs were included, i.e., (a) an exocrine pancreatic sufficient twin pair who is compound heterozygous for p.Phe508del and the class V splice mutation c.3717+12191 C-T which confers some residual wild type CFTR activity (32) and (b) an exocrine pancreatic insufficient twin pair of first cousin parents carrying the ultra-rare class I splice mutation c.3469-2 A-G on both CF alleles. First cousin progeny has been reported to carry homozygous segments in 11% of their genome with an average segment length of 26 cM (33) (Table 1).

**Table 1:** CF twin pair cohort.

**Twin pair**	**Gender**	***CFTR* genotype**	**Age at assessment [yrs]**
53	female	p.Phe508del/p.Phe508del	5
97	female	p.Phe508del/p.Phe508del	9
116	male	p.Phe508del/p.Phe508del	19.8
118	male	c.3469-2 A-G/c.3469-2 A-G	14.9
156	female	p.Phe508del/c.3717+12191 C-T	36.4
180	male	p.Phe508del/p.Phe508del	9.2
237	female	p.Phe508del/p.Phe508del	8.3
238	female	p.Phe508del/p.Phe508del	8.2
244	male	p.Phe508del/p.Phe508del	17.4
291	female	p.Phe508del/p.Phe508del	30.2
292	female	p.Phe508del/p.Phe508del	12.7
294	female	p.Phe508del/p.Phe508del	8.8
309	female	p.Phe508del/p.Phe508del	2
401	male	p.Phe508del/p.Phe508del	9.5
403	male	p.Phe508del/p.Phe508del	6.1
512	female	p.Phe508del/p.Phe508del	5.2

### Determination of TCRß CDR3 Sequence Diversity

DNA was extracted from peripheral blood cells with phenol/chloroform (34) and stored in TE buffer at 4°C. Supplementary Table 1 lists the amount and concentration of DNA in the stock solutions. High-throughput multiplex amplicon sequencing of the CDR3 variable region of the ß-chain of T cell receptors, defined according to IMGT (35) was performed with the immunoSEQ immune profiling system according to the manufacturer's instructions (Adaptive Biotechnologies, Seattle, WA) (36, 37). Barcoded sequencing libraries were prepared from three μg of fragmented DNA by bridge amplification with 15 cycles of PCR. Sequencing of the immune repertoire was performed on an Illumina NextSeq system (Illumina) followed by the data evaluation on the immunoSEQ Analyzer platform (https://clients.adaptivebiotech.com). The amplicon sequences of the TCRβ CDR region obtained for the CF twins were searched for shared sequences of other projects deposited in the immuneACCESS database as of August 6, 2020 and in the VDJdb database as of December 18, 2020 (38).

Diversity of the TCRß sequences was estimated by the measure of Simpson clonality, i.e., the square root of Simpson's index of in-frame productive rearrangements. The generation probability of TCRß sequences was computed with the OLGA algorithm (39). To compare the clonotype V and J gene usage distribution in pairs of individuals, we applied Zvyagin's and coworkers' approach to calculate the Jensen-Shannon distance (21). The Jensen-Shannon divergence (40–42) based on Jensen's inequality and Shannon entropy computes the similarity between two probability distributions, in this case the equally-weighted segment usage in two individuals.

The Jensen-Shannon distance is defined as the Jensen-Shannon divergence divided by the mean entropy of two distributions (21).

[Background (42): $M_{+}^{1}$ (*A*) is a set of probability distributions where *A* is a set provided with some σ-algebra.

Jensen-Shannon divergence JSD: $M_{+}^{1}$ (*A*) x $M_{+}^{1}$ (*A*) → [0, ∞] is a symmetrized and smoothed version of the Kullback-Leibler divergence D (*P* ‖ *Q*). It is defined as

JSD (*P* ‖ *Q*) = ½ D (*P* ‖ *M*) + ½ D (*Q* ‖ *M*) with *M* = ½ (*P* + *Q*)]

For more information please consult the Python package for discrete information theory at https://dit.readthedocs.io/en/latest/measures/divergences/jensen_shannon_divergence.html.

## Results

### CF Twin Clinical Characteristics

**Figure 1:**
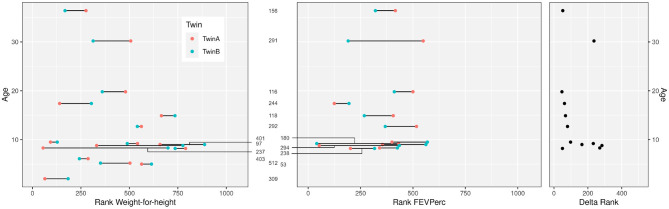
Clinical data of the 16 CF twin pairs discerned by their sample number ID in the European Cystic Fibrosis Twin and Sibling Study (see Table 1). The figure shows the age of the 32 monozygotic twins and their ranks within the 1,060 participants of the European Cystic Fibrosis Twin and Sibling Study for the clinical parameters “Weight-for-height” (left) and “FEVPerc” (middle) at the day of blood sampling. A low rank number indicates a severe outcome. Patients had to be older than 6 years to perform a robust FEV1 measurement. The delta rank (right) indicates the intrapair discordance of the twin pairs. A low delta rank indicates a comparable disease manifestation of the twins. In contrast, large delta values indicate divergent clinical phenotypes.

Ten female and six male monozygotic CF twin pairs aged 2.0–36.4 years (median 9.1 years) were selected for the study (Table 1). The group had on average worse anthropometry and lung function than the dizygous CF sibpairs who had been recruited during the same period from the CF centers. Figure 1 compares the disease severity of study participants within the whole twin and sibpair cohort as indicated by their individual rank for two parameters sensitive for course and prognosis, i.e., weight-for-height and disease centile FEVPerc of the lung function parameter FEV1 (25). The monozygotic twins shared a more similar nutritional and pulmonary status than the CF sibpairs as shown by lower rank number differences in nutritional and pulmonary status (Figure 1). The lower intra-pair difference of the monozygotic twins should reflect their genome, gender and age identity and lifelong sharing of domicile, health care system and local CF care.

### Motivation to Analyze the TCRß CDR3 Region in CF Monozygotic Twins

Each twin pair had been exposed since conception to closely related living conditions including the exposure to antigens. Monozygotic twins share the same MHC I allele profile (43) and the genes coding for the TCR recombination machinery. In analogy to sequence data of healthy monozygotic twins' TCR repertoires (18, 21), we thus expected the sharing of TCR clonotypes and a higher similarity of V and J segment usage in CF monozygotic twins than seen among unrelated CF individuals. By sequencing the variable region of the TCRβ chain with the standardized immunoSEQ protocol developed by Robins et al. (36), we were curious to know whether we could identify a CF-associated signature in the TCR repertoire that is distinct from the sequences of healthy controls (37) stored in the publicly available immuneACCESS database.

TCR ß-chain diversity was assessed in peripheral blood of the 16 CF twin pairs. Table 2 summarizes the features of the 32 CF twins' CDR3 sequence repertoire described in the forthcoming chapters.

**Table 2:** Features of the CDR3 amino acid sequences of the beta chain T cell receptors (TCRß) in monozygotic p.Phe508del homozygous CF twin pairs.

**Sample name**	**Total templates (Sum)^**^a^**^**	**Total productive templates (Sum)^**^b^**^**	**Productive fraction^**^c^**^**	**Rearrangements^**^d^**^**	**Productive rearrangements^**^e^**^**	**Shared NT** **clonotypes (no./%)^**^f^**^**	**Shared AA clonotypes (no./%)^**^g^**^**	**Productiveclonality^**^h^**^**	**Max productive frequency (%)^**^i^**^**
53_A	74,359	60,394	0.8122	58,295	47,444	90/0.16	791/1.67	0.0056	0.13
53_B	54,633	43,850	0.8026	40,931	32,998	90/0.22	791/2.39	0.0086	0.43
97_A	55,365	46,496	0.8398	48,126	40,752	100/0.21	614/1.52	0.0082	0.29
97_B	34,980	29,393	0.8403	30,528	25,923	100/0.33	614/2.4	0.0115	0.85
116_A	22,749	17,803	0.7826	19,238	15,115	13/0.07	126/0.83	0.0102	0.45
116_B	16,668	13,310	0.7985	16,472	13,186	13/0.08	126/0.95	0.0089	0.14
118_A^j^	27,188	22,096	0.8127	22,821	18,477	61/0.27	217/1.17	0.0102	0,35
118_B^j^	24,862	20,075	0.8075	22,178	17,871	61/0.28	217/1.21	0.0086	0.22
156_A^k^	13,389	10,931	0.8164	11,430	9,379	1/<0.01	76/0.83	0.016	0.99
156_B^k^	21,483	17,831	0.83	16,154	13,211	1/<0.01	76/0.58	0.0206	1.14
180_A	9,519	7,653	0.804	8,057	6,399	8/0.09	58/0.91	0.0435	3.03
180_B	19,253	15,353	0.7974	15,764	12,581	8/0.05	58/0.46	0.0339	2.22
237_A	15,161	12,500	0.8245	14,950	12,332	34/0.23	155/1.25	0.0091	0.06
237_B	22,341	18,568	0.8311	21,835	18,162	34/0.16	155/0.85	0.0077	0.12
238_A	52,383	44,480	0.8491	44,272	37,627	36/0.08	684/1.82	0.0068	0.20
238_B	51,082	43,265	0.847	49,265	41,763	36/0.07	684/1.64	0.005	0.05
244_A	17,564	14,737	0.839	16,622	13,977	0	6/0.04	0.0095	0.23
244_B	1,736	1,446	0.8329	1,589	1,321	0	6/0.46	0.0341	1.94
291_A	33,749	26,884	0.7966	30,442	24,532	31/0.10	457/1.86	0.0119	0.57
291_B	43,361	34,600	0.798	35,092	28,112	31/0.09	457/1.63	0.0097	0.38
292_A	83,484	66,033	0.791	59,097	46,808	27/0.05	614/1.3	0.0075	0.48
292_B	38,969	30,983	0.7951	37,940	30,147	27/0.07	614/2.02	0.0059	0.05
294_A	28,112	22,486	0.7999	21,865	17,398	35/0.16	362/2.07	0.049	3.58
294_B	47,793	37,288	0.7802	42,678	33,621	35/0.08	362/1.07	0.0221	2.00
309_A	77,263	60,094	0.7778	62,948	49,303	42/0.07	819/1.66	0.0101	0.68
309_B	50,585	39,846	0.7877	45,482	35,787	42/0.09	819/2.28	0.0061	0.14
401_A	41,606	34,505	0.8293	35,877	30,220	11/0.03	460/1.52	0.0077	0.27
401_B	42,413	35,237	0.8308	37,819	31,687	11/0.03	460/1.45	0.0089	0.48
403_A	12,679	10,791	0.8511	11,916	10,167	44/0.37	336/3.36	0.0104	0.19
403_B	59,694	51,267	0.8588	54,272	46,735	44/0.08	336/0.73	0.0049	0.07
512_A	46,689	37,641	0.8062	42,584	34,320	4/0.01	342/0.99	0.0058	0.06
512_B	30,713	24,890	0.8104	29,149	23,641	4/0.01	342/1.44	0.0074	0.23

a *Number of raw sequencing reads*.

b *Number of in-frame coding reads*.

c *Ratio of in-frame coding reads to raw sequencing reads*.

d *Number of the sequences with rearranged V_β_, D_β_, and J_β_ gene segments*.

e *Number of functional in-frame rearrangements*.

f *The numbers refer to within pair shared clonotypes with identical nucleotide sequence*.

g *The numbers refer to within pair shared clonotypes with identical amino acid sequence*.

h *Square root of Simpson's index of in-frame productive rearrangements*.

i *The maximal frequency of a specific productive rearrangement among all productive rearrangements within a sample calculated as the templates for a specific rearrangement divided by the sum of productive templates for a sample*.

j *First cousin offspring homozygous for c.3469-2 A-G*.

k *Compound heterozygous for p.Phe508del/c.3717+12191 C-T*.

### Frequency Distribution of Clonotypes in the Individual Twins' TCRß CDR3 Repertoire

**Figure 2:**
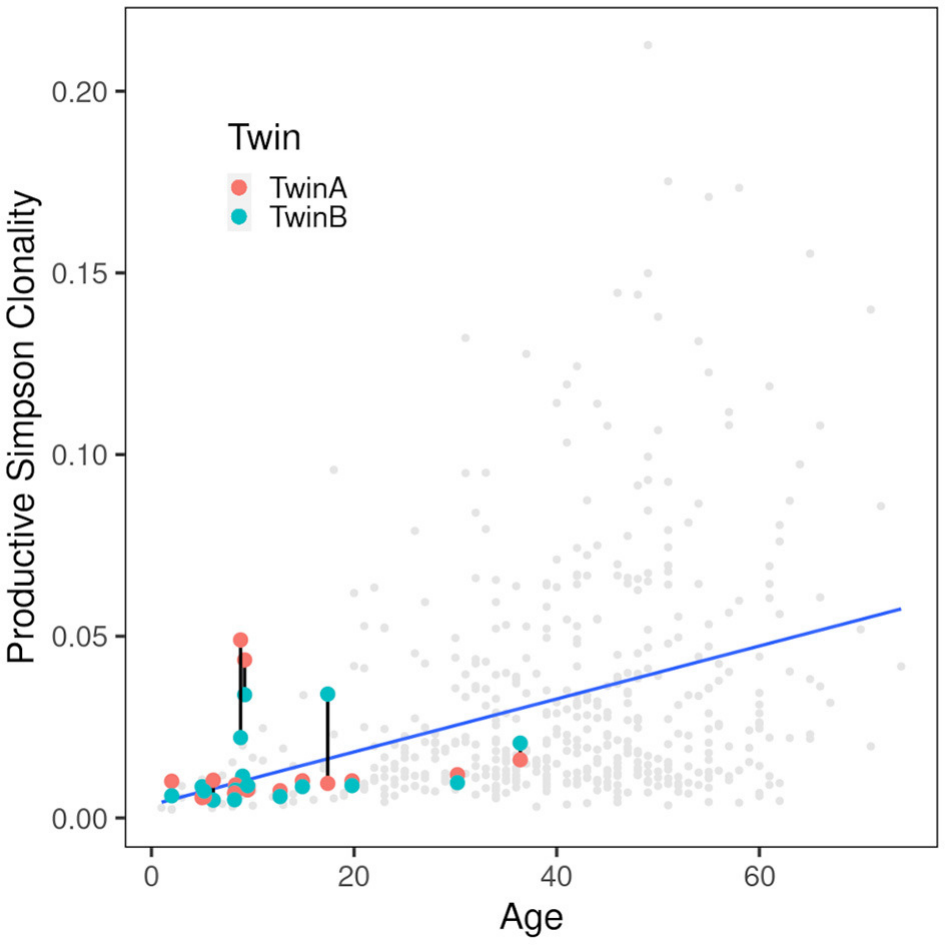
Correlation of productive Simpson clonality with age. Productive clonality is plotted for each twin. Twin pairs are connected with a black line. The gray dots indicate 590 healthy PBMC controls (36, 37) and the blue line indicates the trend of the controls. As expected, the clonality increases with age. Of the twins only the severely affected patients are above the trend line indicating that the CF samples are comparable to those from healthy controls.

Simpson clonality as a measure of the evenness of the distribution of clonotypes varied between 0.0049 and 0.0490 within the range observed for healthy individuals (37) (Table 2). The median value of 0.0095 is lower than that of 0.021 reported for healthy adults consistent with the fact that all but two of our pairs were children or adolescents at the day of sampling and that clonality increases with age reflecting the episodes of clonal expansion due to antigen exposure occurring over time (Figure 2). The low clonality values indicate that the population of TCRß clonotypes is dominated by the naïve T-cell repertoire. Thus CF twins were in general harboring an almost evenly distributed TCRß repertoire.

Five CF patients, two pairs (180, 294) and a singleton (244B), however, showed a higher clonality than expected for their age (Table 2). The singleton belonged to the twin pair with the worst lung function of the cohort and the two pairs represent the twins with the highest intra-pair discordance in lung function, i.e., one patient suffered from an already highly advanced stage of CF pulmonary disease while the other twin's pulmonary function was in the typical CF range of this age. In other words, of the five individuals with the highest Simpson clonality, three belonged to the quartet with the highest annual rate of pulmonary exacerbations and the other two individuals were the other monozygotic twin. These five patients harbored the within sample most abundant clonotypes that made up 2–4% of their individual TCRß repertoire (Table 3). Of these eight sequences, two sequences were not present in the immuneACCESS database, three sequences were detected at a frequency of <1% and another three sequences were common clones identified in 7–11% of samples deposited in the database (Table 3). In summary, the individually most abundant clonotypes in our data set represent an ensemble of private, rare and common public clones.

**Table 3:** The most frequent non-shared TCRß clonotypes in the CF twin samples.

**Sample**	**CDR3 amino acid sequence**	**Count**	**Frequency in** **twin sample (%)**	**Presence in immuneACCESS database**	
				**No of samples**	**Frequency (%)**
294_A	CASSLPREGNEQYF	804	3.58	4	0.04
294_A	CASSPGDTIYF	443	1.97	662	7.04
180_A	CASSYHRSTAEAFF	232	3.03	0	0
180_A	CASSFAGALQETQYF	150	1.96	63	0.67
180_B	CASSLWGWEQYV	341	2.22	0	0
180_B	CASSLRGGSYEQYV	324	2.11	55	0.59
294_B	CASSLSRGEAFF	745	2	718	7.64
244_B	CASRQGSQPQHF	28	1.94	1051	11.18

### Intrapair Sharing of Clonotypes at the Nucleotide and Amino Sequence Levels

**Figure 3:**
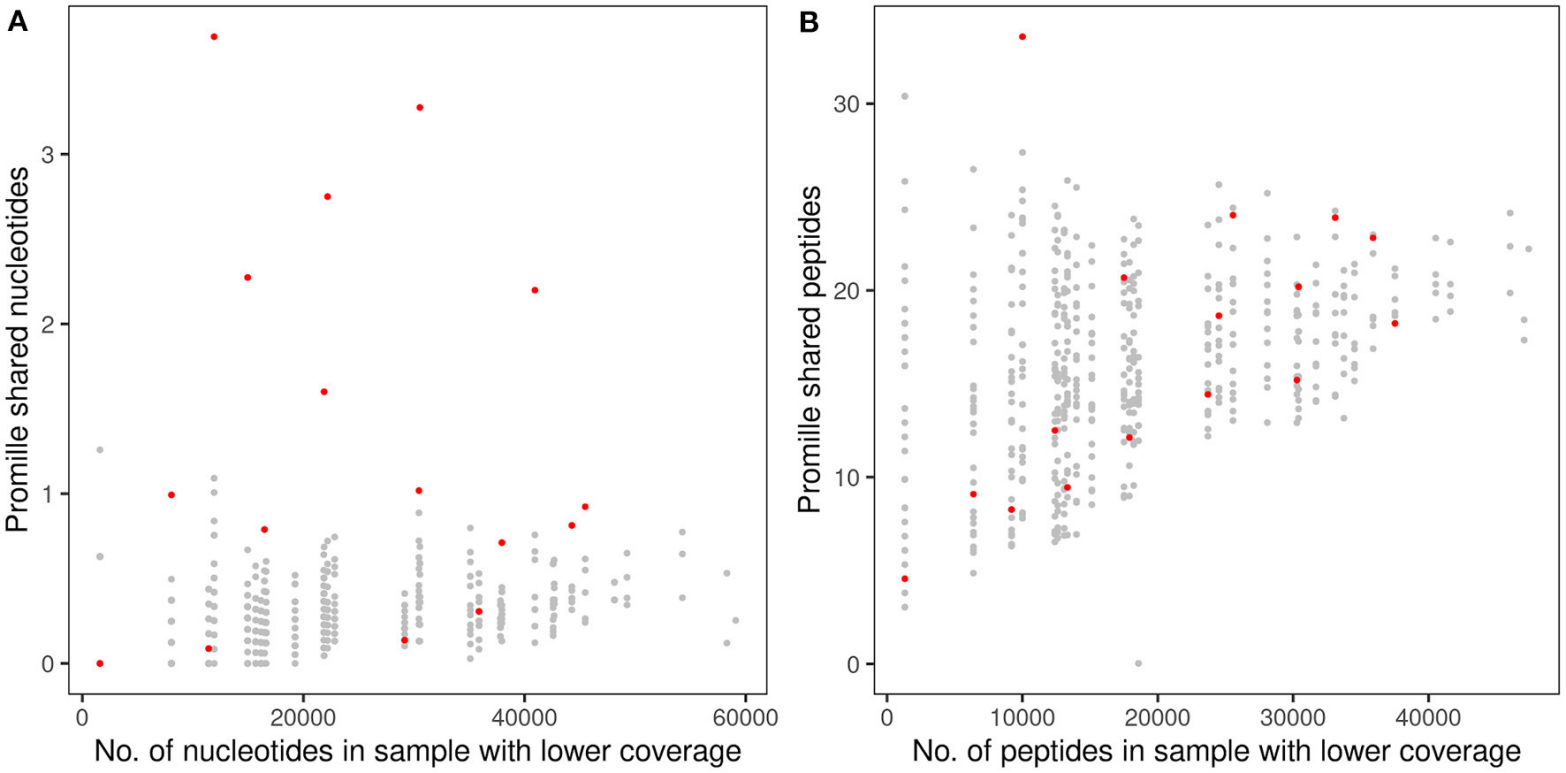
Proportion of shared TCRß CDR3 clonotypes in monozygotic CF twins at the nucleotide sequence **(A)** and amino acid sequence **(B)** levels. The abscissa indicates the numbers of raw sequencing reads **(A)** and in-frame coding reads derived peptides **(B)** of the twin's sample with the lower number of reads that were taken as the reference for the calculation of the proportion of shared clonotypes. The dots indicate the proportion of CDR3 sequences shared within the 16 CF twin pairs (red) or between 480 arbitrary pairs of unrelated CF twins generated from the same data-sets (gray). Please note the different scales of the ordinate in **(A,B)**.

A twin shared on the average about 30 clonotypes of identical nucleotide sequence with the twin sibling (inner quartiles: 11–42; range 0–100) (Table 2). Intra-pair sharing occurred for in total 0.11% of all CDR3 sequences. For comparison, we determined the sharing of clonotypes within CF twin pairs arbitrary pairs of unrelated CF twins generated from the same data-sets (Figure 3A; Supplementary Table 2). The inter- vs. intrapair comparison revealed that the number of shared nucleotide sequences was significantly higher within monozygotic CF twin pairs (*P* = 0.000026, Mann-Whitney rank test). The almost identical genomic blueprint and the exposure to the same environment since conception had led to a small repertoire of shared TCRß clonotypes with identical nucleotide sequence. Within our small cohort we did not identify any association between the number of shared CDR3 sequences and age, concordance and severity of CF disease.

Next, we investigated the sharing of TCRß clonotypes at the amino acid sequence level in our CF twin data set. The CF twins did not share CDR3 peptides more often than arbitrary pairs assembled from the 32 CF twins (*P* = 0.766) (Figure 3B; Supplementary Table 2). The numbers of shared amino acid sequences increased with cloneset size consistent with previous reports on healthy monozygotic twins (21).

**Figure 4:**
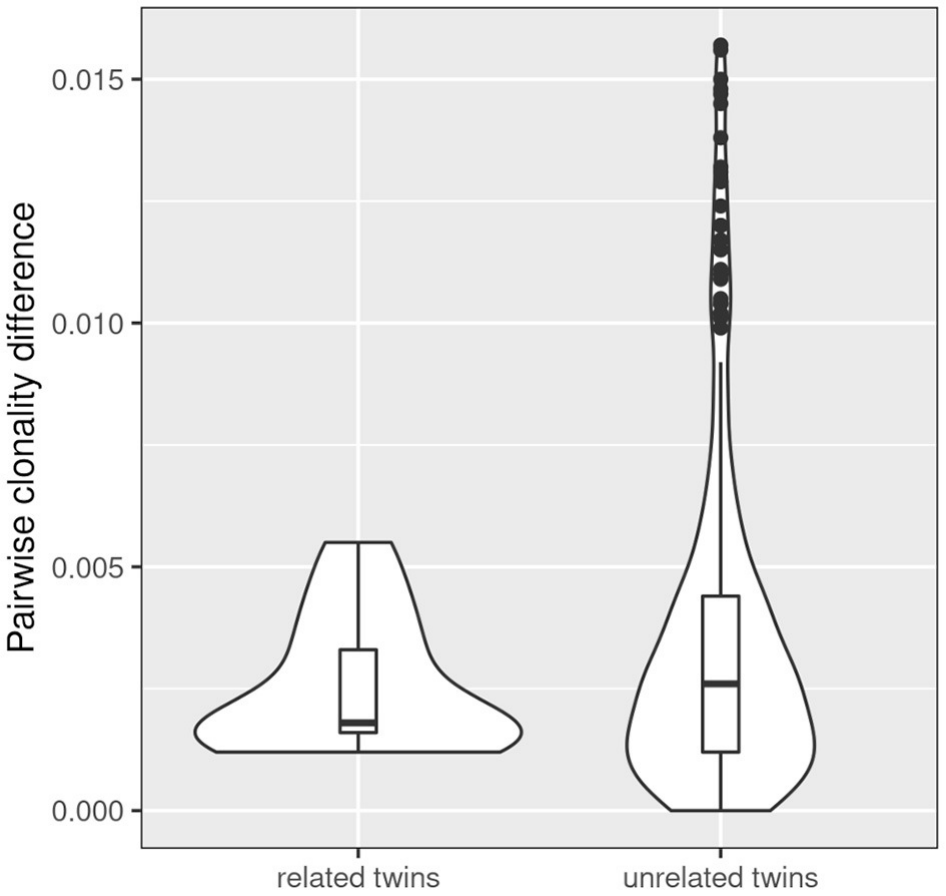
Comparison of intra- and inter-pair clonality difference. The absolute difference in clonality was calculated for each related (*n* = 13) and unrelated twin pair (*n* = 312). The three severely affected outlier pairs 180, 244, and 294 were excluded from the analysis. A non-significant trend was observed that twin pairs show a lower clonality difference than unrelated patient samples.

Simpson clonality is a statistic measure of how much of the repertoire is made up of expanded clones (see above). The intra-pair variation of Simpson clonality of the CF twins was low (median 0.0019, range 0.0012–0.0055). We observed a non-significant trend that twin pairs show a lower clonality difference than unrelated patient samples (Figure 4) suggesting that genetic constraints and the shared environmental exposure may favor a higher similarity of the TCRß repertoire.

### Intrapair Sharing of V and J Gene Segment Usage in CF Twins

**Figure 5:**
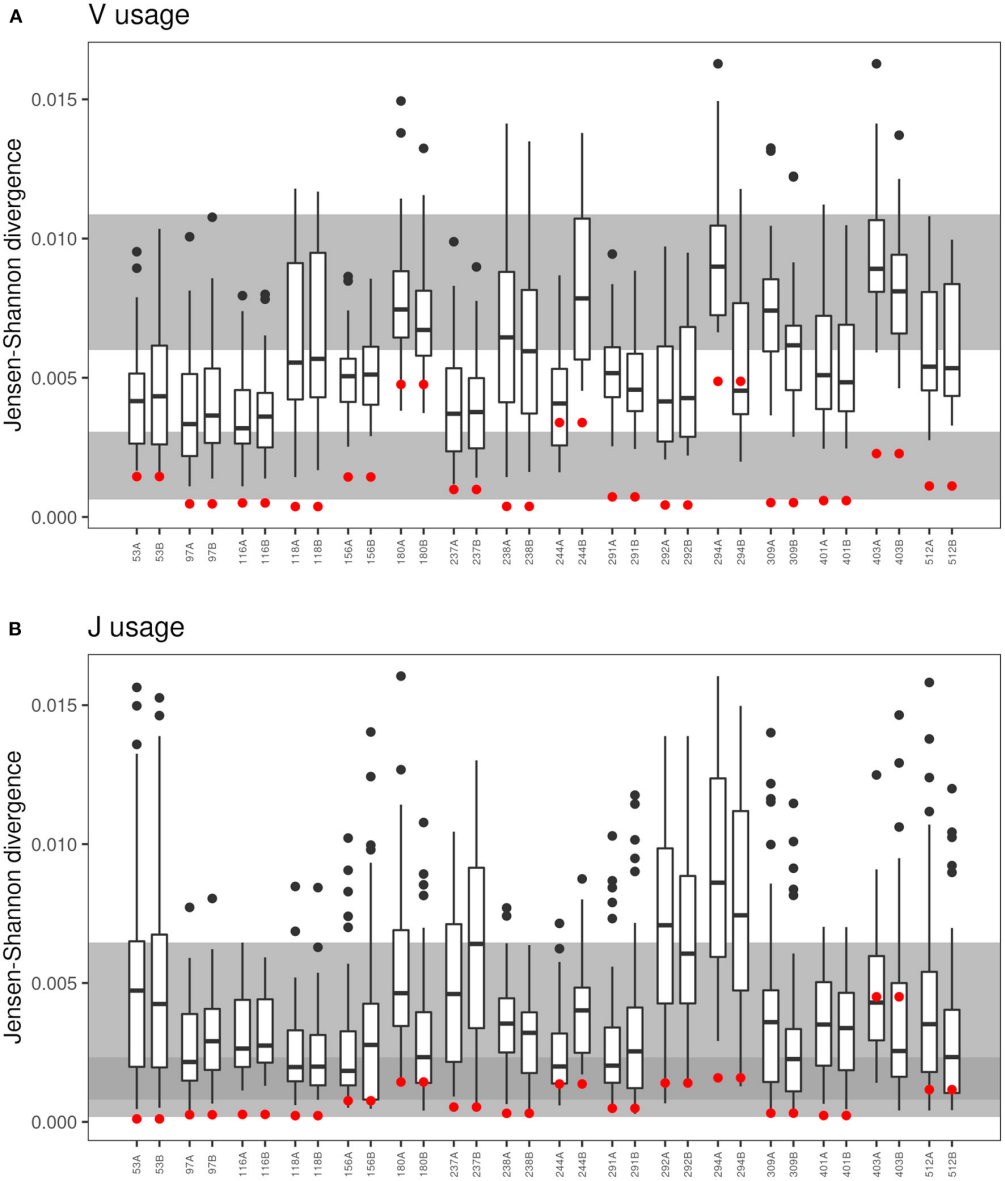
Comparison of VJ-usage in related und unrelated twin pairs. The boxplot presentation shows the Jensen-Shannon divergence (40–42) for the comparison of each sample to the twin sibling and to the 30 unrelated twins for the **(A)** V-segment and **(B)** J-segment usage of the CDR3. Red dots represent the Jensen-Shannon divergence to the twin sibling. The gray background areas indicate the reference values for healthy unrelated twins (upper gray panel) and related healthy twins (lower gray panel) (21). In case of the J-segment, the reference panels overlap indicated by the dark gray area.

Monozygotic twins share the TCR recombination machinery. Hence, based on data published on healthy twins (18, 21), we expected a more similar distribution of V and J gene segments within a CF twin pair than between unrelated CF individuals. The Jensen-Shannon divergence divided by mean entropy (21, 42) was calculated as an equally weighted measure of the similarity of the TCRß populations of V and J gene usage for in-frame TCRß clonotypes of all possible pairs of individuals (Figure 5). The shortest Jensen-Shannon distance between the populations of V and J gene segments was mutually to the other CF twin individual for 13 of 16 twin pairs in case of the V usage and for 8 of the 16 twin pairs in case of the J usage. This data verify the observation in healthy monozygotic twins (21) that genetic constraints are involved in the selection of V and J gene segments.

In contrast to the observation in the majority of twin pairs, individual signatures of V and J usage were also noted. In case of the five pairs 180, 244, 291, 292, and 294 the J sequence population was most similar from one twin to the twin sibling, but not vice versa, and both twins of the pairs 156, 403, and 512 shared a more similar distribution of J sequences to unrelated CF individuals than to the other twin. Similarly, the twin pair 180 and the twins 244A and 294B had a more similar V usage with unrelated CF patients than with their twin sibling. These three twin pairs also demonstrated the most uneven distribution of clonotypes and the poorest age-corrected lung function (see Figure 1).

**Figure 6:**
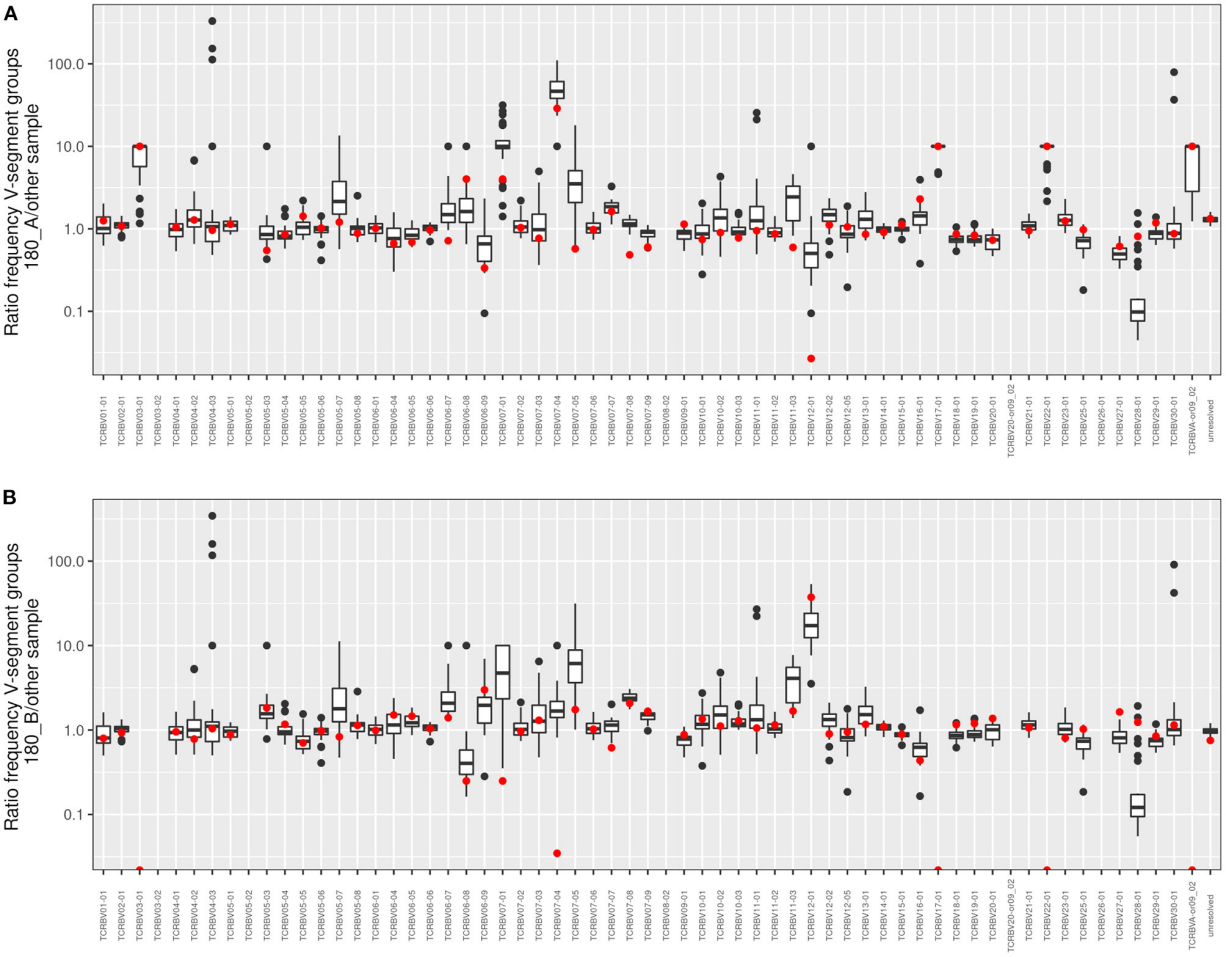
Frequency comparison of V-segment usage of the distant twin pair 180. The boxplots show pairwise comparisons of both **(A)** twin 180_A and **(B)** twin 180_B to the twin sibling and the 30 unrelated twins for all V-segment groups. Red dots symbolize the comparison to the 180 twin sibling. Values above 1 indicate an overrepresentation of the frequency of the respective V-segment family in the investigated twin sibling. If no clonotypes were found in one of the “other” samples the value was fixed to 10 to allow a calculation and to take into account that the V-segment family occurs in the 180_A or 180_B sample, respectively.

Figure 6 visualizes the individual V segment usage in the 180 twin pair who for his age was most affected by severe pulmonary disease in our cohort. The box plots depict the ratio of copy numbers of V segments of twin 180A or twin 180B to those of all other members of the CF twin cohort. Of the 63 V segment classes, the usage was similar within a factor of ±2 for about 80% of classes among all study participants. However, four V gene segments were more than 2-fold more frequently detected in the TCRß chain repertoire of both twins than in the unrelated individuals. Moreover twin 180A harbored nine segments and twin 180B carried five segments two- to more than hundred fold more frequently in his TCRß repertoire than all other study participants including his twin brother. In summary, twins 180A and 180B exhibited a more individual and more skewed usage of V gene segments than all other examined monozygotic CF twins.

### Sharing of Clonotypes at the Amino Acid Sequence Level in the CF Twin Cohort

**Figure 7:**
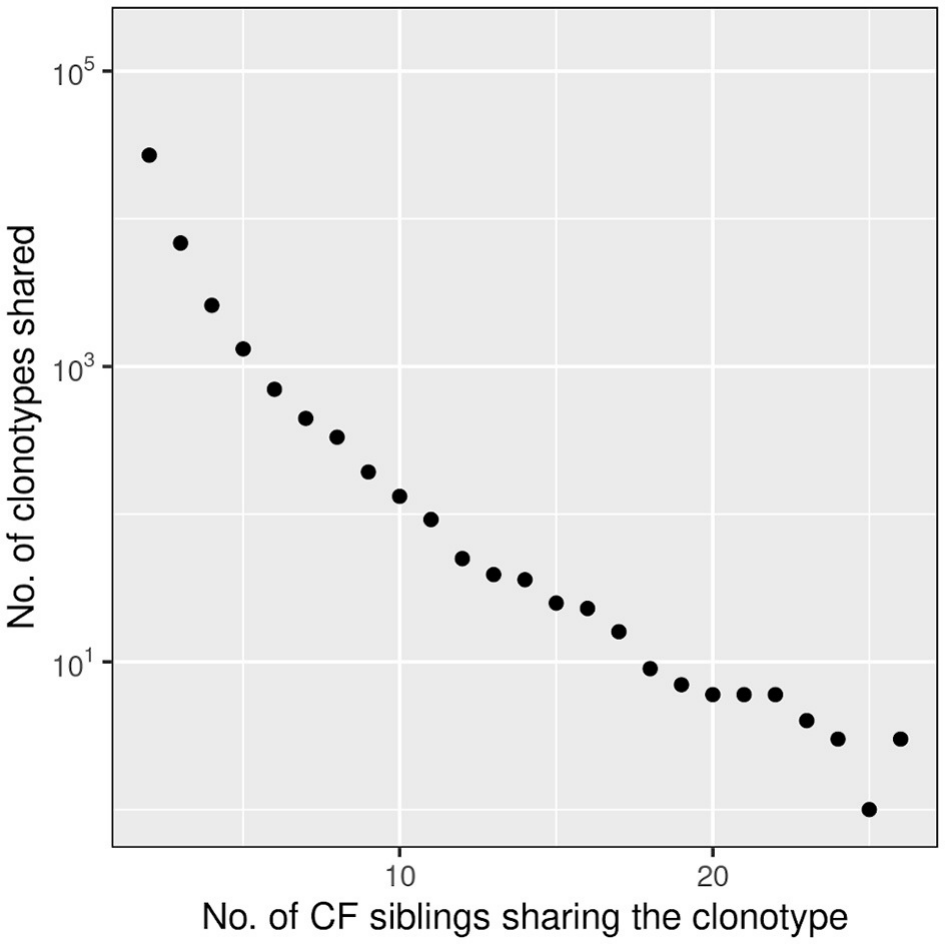
Frequency distribution of amino acid clonotypes present in two or more CF siblings. The frequency distribution of shared amino acid clonotypes (*n* = 39,812) shows that the majority is found in two samples. However, there are also public clonotypes which are present in more than 50% of the twins. In this presentation we did not distinguish between related and unrelated twin pairs.

39,812 clonotypes were detected twice of more in our data-set. Figure 7 depicts the number of shared clonotypes in the CF twin samples. The frequency of detection of the presence of a sequence in the sample set shows a concave deviation from a logarithmic decline consistent with our knowledge that the generation probability of human CDR3 amino acid sequences varies over 20 orders of magnitude (39) and that the most common sequences are present in the TCRß repertoire of virtually any individual.

By utilizing the OLGA algorithm recently published by Sethna and colleagues (39), we calculated the probability of generating CDR3 amino acid sequences for various subsets of shared clonotypes. Of the CDR3 amino acid sequences exclusively identified in both siblings of one twin pair, the median probability of generating one of these clonotypes was computed to be 3.1 × 10^−8^ [inner quartiles 8.4 × 10^−9^-7.9 × 10^−8^; range 2.5 × 10^−21^-2.6 × 10^−6^]. A 2-fold higher median generation probability of 6.7 × 10^−8^ [2.3 × 10^−8^-1.6 × 10^−7^; 0-8.1 × 10^−6^] was calculated for clonotypes that were only detected in unrelated patients and a 10-fold higher median probability of 2.8 × 10^−7^ [1.2 × 10^−7^-6.0 × 10^−7^; 3.1 × 10^−15^-6.8 × 10^−6^] for the sequences identified in one to 12 twin pairs and singletons of the remaining pairs. Since generation probabilities larger than 10^−10^ (44)−10^−8^ (45) have been estimated to be present in any individual we can safely assume that at least 75% of the detected shared clonotypes in our CF twin cohort are public clones commonly present in the human TCR repertoire.

### Abundance of CF Twin Shared Amino Acid Sequence Clonotypes in Health and Disease

**Figure 8:**
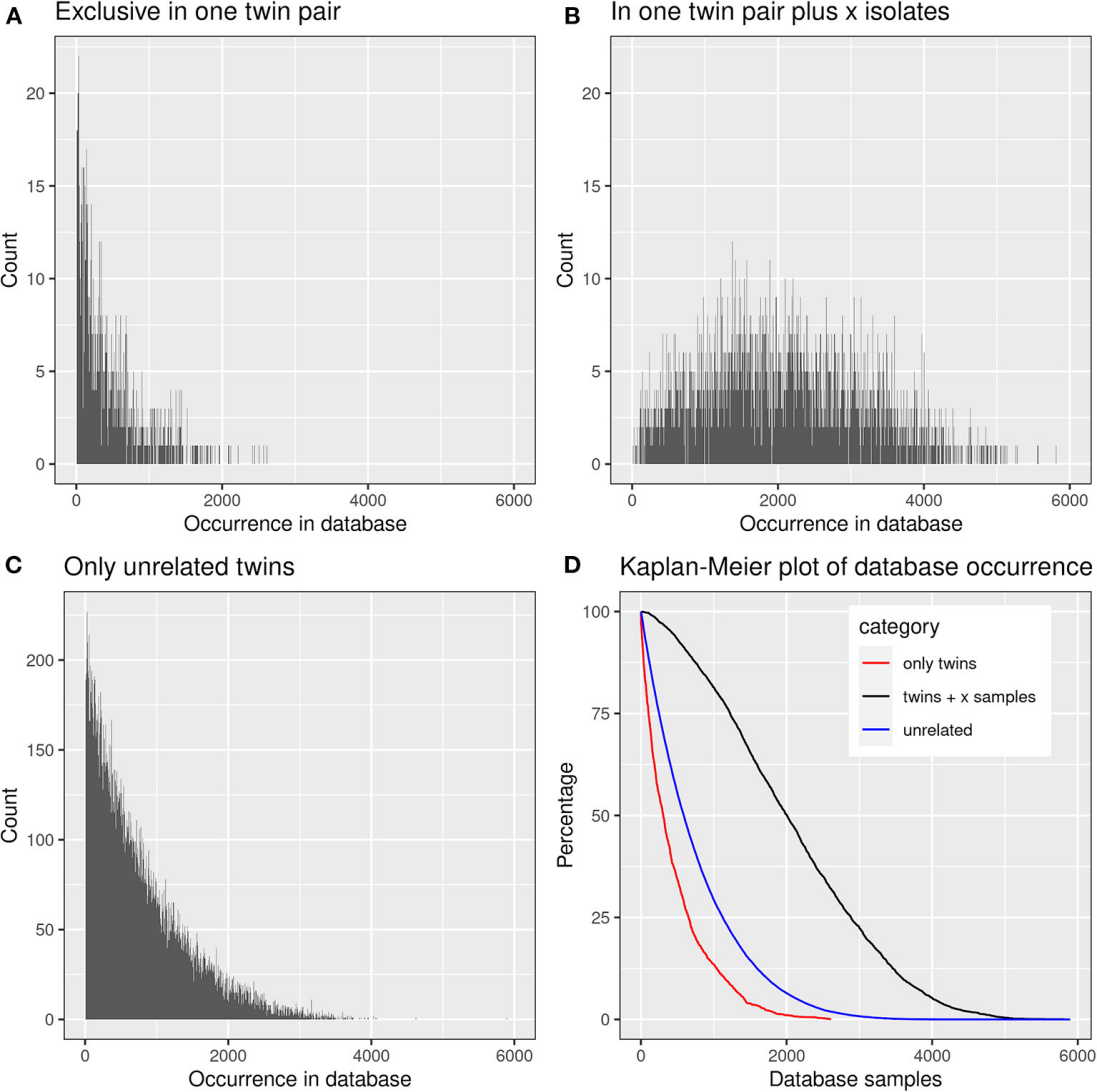
Presence of frequent amino acid clonotypes of CF siblings in the samples deposited in the immuneACCESS database. 39,812 amino acid clonotypes were detected in at least two samples from CF siblings. **(A)** 1,384 clonotypes exclusively identified in a related twin pair were found in an only low number of database samples. **(B)** 3,430 clonotypes were detected in at least one related CF twin pair plus CF singletons. These public clones are very frequent within the immuneACCESS database. **(C)** 34,998 clonotypes were only detected in unrelated CF siblings. The majority of these clonotypes is only present with low frequency in the immuneACCESS database. **(D**) The Kaplan-Meier plot shows that clonotypes present in twins and at least one independent sample are more frequently found in the immuneACCESS database (black line). In contrast, clonotypes only present in twins or in unrelated samples are comparably rare.

We were curious to know whether and to which extent the shared CDR3 sequences had already been detected in healthy individuals or patients with other diseases. The screening of the TCRß repertoire of 9,100 samples in the immuneACCESS database revealed that 39,739 clonotypes, i.e., 99.82% of total, had already been previously found in 1–5,896 samples (median: 587) (Figure 8).

Just 73 shared clonotypes had not been detected before in the other projects, 31 of which in a twin pair and 42 in unrelated CF patients. Their median generation probability was computed to be 7.8 × 10^−11^ (inner quartiles 1.2 × 10^−11^-2.9 × 10^−10^; range 2.5 × 10^−21^-2.8 × 10^−8^). These values are substantially lower than calculated for the three sets of shared clonotypes described in the previous paragraph. Hence these shared CF twin clonotypes that yet have not been documented in the immuneAccess database are indeed not common in the human TCRß repertoire. A subset of these sequences may have emerged in the monozygotic CF twins during shared exposure to opportunistic pathogens as a consequence of their inherited susceptibility to infection. Consistent with this interpretation none of these 73 clonotypes was listed in the VDJdb database (38) that contains clonotypes with known specificities. Conversely, 47 of the 84 clonotypes that were identified in more than half of the CF twin samples could be mapped to the epitopes listed for *Mycobacterium tuberculosis* (one entry) and one or more viruses (67 entries) in the VDJdb database (38). Forty clonotypes were assigned to CMV epitopes and 11 clonotypes to EBV epitopes, eight of which were mapped to both CMV and EBV epitopes. These 84 most abundant clonotypes in the CF twin cohort have a median generation probability of 1.4 × 10^−6^ [8.3 × 10^−7^-2.1 × 10^−6^; 2.7 × 10^−7^-5.6 × 10^−6^] demonstrating that these CDR3 sequences present public clones of the non-CF community.

The three data subsets of shared clonotypes of our CF cohort (twin pair only, unrelated patients only, twin pairs and unrelated patients) were analyzed for their frequency distribution among the non-CF samples of the immuneACCESS database (Figure 8, Table 4). Interestingly, the empirical frequency distributions of the three subsets in the database matched with their distribution of TCRß generation probabilities predicted by the OLGA algorithm. If a clonotype had only been identified in the two sibs of a twin pair, it generally was infrequently present in the non-CF samples. 85% of the clonotypes were detected at a frequency of <10% in the immuneACCESS database. Similarly, clonotypes solely identified among unrelated CF patients were mainly detected at low frequency in the non-CF samples (Table 4). However, they were more common than the twin pair sequences indicated by a smaller portion of 68% of sequences that were identified in <10% of non-CF samples. The frequency distribution of clonotypes from both these CF data sets was exponentially declining in the non-CF sample collection (Figures 8A,C). Conversely, if a clonotype had been identified in the two siblings of a twin pair and in addition in unrelated CF patients, the frequency among non-CF samples showed a skewed Gaussian distribution (Figure 8B). This sub-set of CF clonotypes mainly consists of widely distributed public clones (Table 4), i.e., a majority of 83% of the CF clonotypes were present in more than 10% of the non-CF samples. Since these frequent CDR3 sequences are shared by both monozygotic twin pairs and unrelated individuals, we hypothesized that they should represent a pool of sequences that are non-randomly selected by both global and individual genetic and environmental factors. The OLGA algorithm predicted a high median generation probability of 2.8 × 10^−7^ for this subset of CF clonotypes consistent with the empirical finding that the most abundant TCRβ CDR3 sequences arise from the higher generation probability compared to the repertoire median (46). These conserved public sequences are central within TCR sequence-similarity networks (47, 48).

**Table 4:** Presence of the TCRß clonotypes found in two or more CF samples in the 9,100 non-CF samples of the immuneACCESS database.

**Sharing of clonotypes in the CF twin data set**		**Frequency (%) in non-CF samples**
	**Total no. of clonotypes**	**median (inner quartiles; range)**
Exclusive in one twin pair	1,384	3.2 (1.1–7.0; 0–27.8)
One twin pair + unrelated patients	3,430	21.3 (13.0–29.4; 0.05–61.9)
Only unrelated patients	34,998	6.3 (2.6–11.9; 0–62.7)

**Figure 9:**
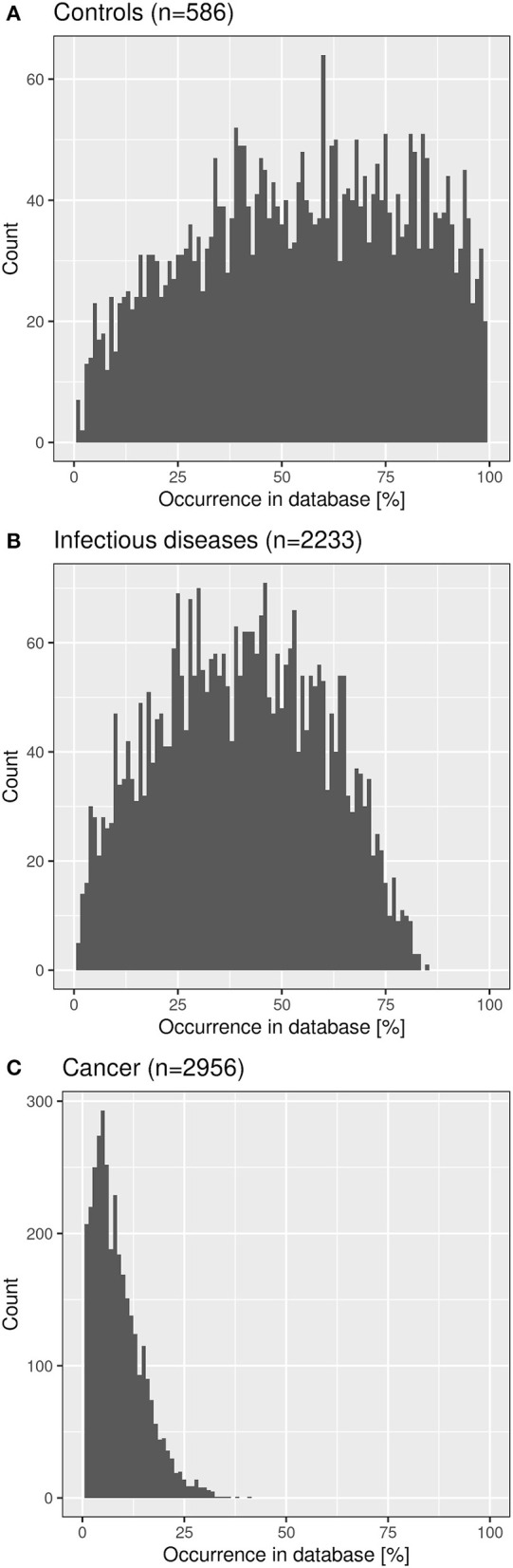
Frequency distribution of shared CF amino acid clonotypes in samples of healthy humans **(A)**, patients with infectious disease **(B)** and patients with cancer **(C)** deposited in the immuneACCESS database.

We checked the distribution of the “public” CF clonotypes (Figure 8B) in subsets of the immuneACCESS database, i.e., samples from healthy controls, patients with infectious disease or cancer. As seen in Figure 9, the largest abundance of these “public” CF clonotypes were observed among healthy non-CF people, less represented in individuals with infectious disease and uncommon in patients with cancer. Hence we would like to conclude that the clonotypes that are shared within twin pairs and between unrelated CF sibs represent public clones that are common in healthy humans. We speculate that in humans – at least in those of European descent – a subportion of the naïve T cell receptor repertoire is overrepresented that is not necessarily associated with disease as it had been deduced from murine animal models (49, 50).

## Discussion

This study examined the number of unique TCRß sequences in peripheral blood specimens collected from monozygotic p.Phe508del homozygous CF twins. In contrast to expectation we identified only a few yet unknown CDR3 sequences at amino acid sequence level. More than 99% of sequences had already been observed in healthy people or other diseases. The recurrent airway infections which had affected our twin cohort since early childhood had not introduced any disease-specific bias in the TCRß repertoire. We predominantly examined CF children and adolescents who were carrying individual CDR3 sequences at low copy numbers. Individual clonal expansions were primarily seen in the clinically most severely affected patients suggesting that clonality may be higher in CF adults with more advanced lung disease. Alternatively, the compartmentalization in CF airways, with neutrophils accumulating in the lumen, whereas T cells stay in the submucosa and lymph nodes and are excluded from the lumen (9), could prevent a more active role of T cells against the luminal pathogens and the neutrophil-driven chronic inflammation.

To our knowledge TCRß clonotypes were investigated in the yet largest cohort of monozygotic twins in humans with a monogenic life-limiting disease. Previous studies in healthy adults (18, 21, 51) have shown a genetic influence on the usage of V and to a lesser non-significant extent on the usage of J gene segments. In contrast we observed a similar genetic influence on the usage of both V and J segments in our monozygotic CF twin cohort which just may be the consequence of the fact that the previous analyses examined only three (21), five (51) or six pairs (18) whereas this study is based on 16 twin pairs. Since no genetic impact of gene segment pairs for both variable and joining regions beyond that for gene segment usage has been identified in TCR α-β pairs (18), we would like to conclude in accordance with the literature that recombination accounts for the genetic influence of V and J gene segment usage. This genetic bias showed up in the Jensen-Shannon distance of V and J segment usage that was typically shortest to the twin individual (Figure 7). Exceptions were the clinically most discordant and/or most severely affected twin pairs where clonal expansion probably caused by recurrent pulmonary infections overshadowed the impact of the identical genomic blueprint. Recently the T-cell receptor repertoire has been reported for 28 monozygotic twin pairs of whom one or both twins were affected by the polyfactorial inflammatory bowel disease (IBD) (52). The Jensen-Shannon divergence of the V gene usage of TCRß was substantially higher in the IBD than in the CF twin cohort which we would not ascribe to the diverse disease etiologies but rather to the age difference that the IBD patients were mid-age adults and all but two CF twin pairs were children and adolescents.

Intrapair sharing of clonotypes was significantly more frequent among monozygotic CF twins than among pairs of unrelated CF patients. Complete nucleotide sequence identity was observed in about 0.11% of CDR3 sequences. Deep sequencing of the TCR repertoire in cord blood and healthy twins and unrelated individuals of different age (23) have provided convincing evidence that a large portion of these shared clones are derived from the same progenitor T cells generated during fetal development. However, besides the persistence of fetal clones further mechanisms should exist that generated an increased repertoire of shared clonotypes among the monozygotic CF twin pairs.

More than 99.8% of the clonotypes of the CF twin cohort had already been detected at amino acid sequence level in a cohort of about 10,000 non-CF individuals whose TCRß repertoire had been investigated by matching protocols of CDR3 amplicon sequencing. The subset of shared clonotypes in the CF cohort showed divergent frequency distributions in the non-CF cohort. Clonotypes were typically re-identified in only few non-CF individuals if the sequence was shared by A) either the sibs of a twin pair or B) two or more unrelated CF patients (cf. Figure 8). The sharing of clones in these two scenarios can be ascribed to either identical genetic makeup including MHC I alleles and shared environmental exposure in case of the monozygotic CF twins (A) or to positive selection of a functional and self-tolerant T cell repertoire driven by recombination machinery, structural and functional constraints (15) in case of unrelated CF patients (B). In both scenarios (A) or (B) the frequency distribution of clonotypes in the non-CF cohort showed an exponential decline indicating that the probability is comparatively low that the same clonotype will re-emerge in an unrelated individual. In contrast, if both scenarios (A) and (B) applied, the respective clonotypes were widely distributed in the non-CF cohort (cf. Figure 8B). These CDR3 sequences are public clones in the narrow sense which survive thymic selection in healthy humans with high probability (Figure 9). The co-occurrence in monozygotic twins and unrelated individuals suggests that these clonotypes are positively selected by both genetic and functional factors. Public clones have been frequently detected in mouse models of autoimmune and infectious diseases, but the immuneACCESS database tells us that these clones are characteristic for healthy humans.

In conclusion, this study uncovered in general a low Simpson clonality indicating that the population of TCRß clonotypes of the CF twins was dominated by the naïve T-cell repertoire. The genetic constraints of TCR repertoire formation showed up in highly similar distributions of the usage of J and V genes of in-frame clonotypes in a CF twin pair. Almost all CDR3 peptides shared by CF twins were public clones prevalent in health and disease, but the few shared CDR3 amino acid sequences that were found for the first time, probably represent CF – associated clonotypes.

## Data Availability Statement

The original contributions presented in the study are publicly available. This data can be found here: https://clients.adaptivebiotech.com/pub/fischer-2021-fi.

## Ethics Statement

The studies involving human participants were reviewed and approved by Ethics Committee of Hannover Medical School (no. 1300 and no. 3739, last update May 8th, 2014). Written informed consent to participate in this study was provided by the participants' legal guardian/next of kin.

## Author Contributions

FS and BT planned the study. SF and BT analyzed the data. SF and BT wrote the manuscript. All authors contributed to the article and approved the submitted version.

## Conflict of Interest

The authors declare that the research was conducted in the absence of any commercial or financial relationships that could be construed as a potential conflict of interest.
